# Role of Transcatheter Arterial Embolization in Acute Refractory Non-variceal Upper Gastrointestinal Bleeding Not Controlled by Endoscopy: A Single-Center Experience and a Literature Review

**DOI:** 10.7759/cureus.29962

**Published:** 2022-10-05

**Authors:** Charbel Ishak, Haider Ghazanfar, Sameer Kandhi, Ahmed Alemam, Hafsa Abbas, Harish Patel, Sridhar Chilimuri

**Affiliations:** 1 Interventional Radiology, BronxCare Health System, Affiliate of Icahn School of Medicine at Sinai, New York, USA; 2 Internal Medicine/Gastroenterology, BronxCare Health System, New York, USA; 3 Internal Medicine, BronxCare Health System, New York, USA

**Keywords:** endovascular embolization, refractory upper gastrointestinal bleeding, anemia, morbidity, mortality, upper endoscopy, interventional radiology guided embolization

## Abstract

Introduction

Acute upper gastrointestinal bleeding (UGIB) is a medical emergency and a common cause of hospital admissions worldwide. It has traditionally been treated with resuscitation and endoscopic intervention as the first-line therapy. In this study, we assessed the adjunctive role of transcatheter arterial embolization (TAE) in patients with uncontrolled UGIB after an endoscopic intervention.

Material and methods

A retrospective chart review of patients requiring TAE of UGIB which was not controlled by endoscopic intervention in BronxCare Health System from 2018 to 2021 was done. Patients who were more than 18 years of age and required TAE during the time period of the study were included in the study. Patients' charts were reviewed for patients' demographics, comorbidities, hospital course, imaging findings, esophagogastroduodenoscopy findings and intervention, and interventional radiology intervention and clinical outcome.

Results

A total of 10 patients were included in the study. A majority of the patients were male. Transcatheter atrial embolization was successful in all the 10 patients. Coils were used in seven patients while particulate polyvinyl alcohol 500 micron particle was used in two patients and vascular plug was used in two patients. Out of the 10 patients, four expired during the hospital course. None of the patients died secondary to UGIB. Three of the patients expired due to severe sepsis with septic shock secondary to pneumonia while one patient died because of respiratory failure due to lung collapse secondary to endobronchial lesion.

Conclusion

Refractory acute UGIB is associated with significant morbidity and mortality. TAE is a minimally invasive measure that should be considered early in the treatment of UGIB which is refractory to conventional endoscopic management. Our case highlights the importance of TAE in a patient with refractory UGIB after endoscopic intervention.

## Introduction

Acute upper gastrointestinal bleeding (UGIB) is a medical emergency and a common cause of hospital admissions worldwide. It has traditionally been treated with resuscitation and endoscopic intervention as the first-line therapy. In 70-75 % of cases, UGIB can cease spontaneously with no interventions. Despite recent advances in clinical medicine, acute UGIB is associated with an average mortality rate of 13.8% and is a significant contributor to healthcare costs in the United States of America [1]. The mortality rate increases proportionally with the patient’s age.

Bleeding originating from the gastrointestinal tract above the ligament of Treitz is termed as UGIB while bleeding originating below the ligament of Treitz is termed as lower gastrointestinal bleeding (LGIB). UGIB usually presents as hematemesis, coffee ground emesis, or melena. In the case of massive UGIB, the patient can also present with hematochezia. According to a meta-analysis, history of melena (likelihood ratio [LR]: 5.1-5.9), melenic stool on examination (LR: 25), nasogastric lavage with blood or coffee ground material (LR: 9.6), and serum urea nitrogen:creatinine ratio of more than 30 (LR: 7.5) were associated with an increased likelihood that the patient was having a UGIB [2]. Common causes of nonvariceal UGIB include peptic ulcer disease, gastric erosions, esophagitis, arterio-venous malformations, Dieulafoy's lesions, Mallory-Weiss tears, and benign and malignant tumors among other uncommon causes [3,4]. About half of the cases of acute UGIB can be attributed to peptic ulcer disease [3]. In our retrospective study, we assessed the adjunctive role of transcatheter arterial embolization (TAE) in patients with uncontrolled UGIB after the endoscopic intervention.

## Materials and methods

A retrospective chart review of patients requiring TAE of UGIB which was not controlled by endoscopic intervention in BronxCare Health System, New York, from 2018 to 2021 was done. Adult patients with UGIB who failed endoscopic intervention and subsequently required transcatheter embolization were included in this study. Patients with LGIB and pregnant patients were not included in the study. The patient’s chart was reviewed for demographics, comorbidities, hospital course, imaging findings, esophagogastroduodenoscopy (EGD) finding and intervention, and interventional radiology intervention and clinical outcome. Images of endoscopic findings, computed tomography findings, and interventional radiology findings and intervention were included in the study. This study protocol was reviewed and approved by the Institutional Review Board of BronxCare Health System, with approval number 02 10 22 01.

## Results

A total of 10 patients were included in the study. A majority of the patients were male. TAE was successful in all 10 patients. Coils were used in seven patients while particulate polyvinyl alcohol (PVA) 500 micron particle was used in two patients and vascular plugs were used in two patients. Out of the 10 patients, four expired during the hospital course. None of the patients died secondary to UGIB. Three patients expired due to severe sepsis with septic shock secondary to pneumonia while one patient died because of respiratory failure due to lung collapse secondary to an endobronchial lesion. A summary of the hospital course of the 10 patients has been presented as follows.

Case 1 

**Figure 1:**
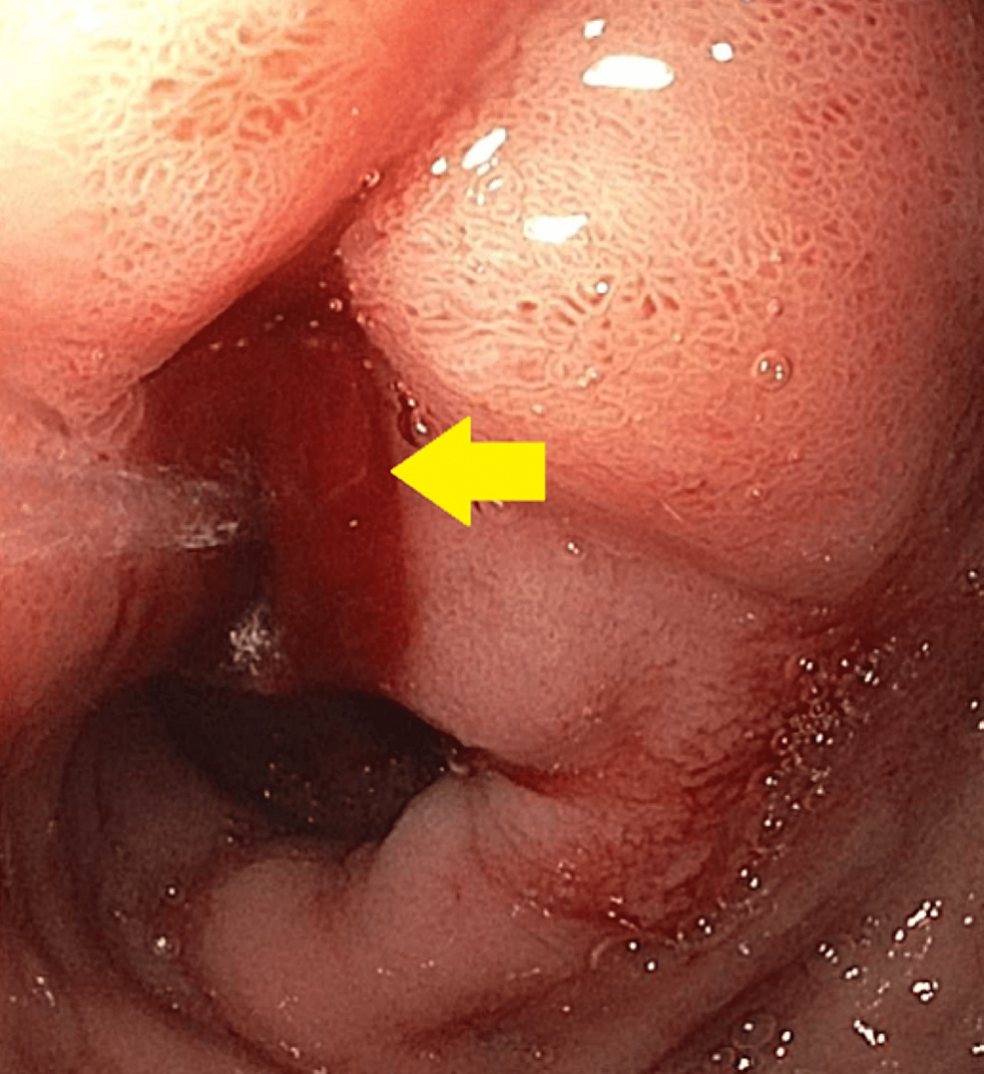
Esophagogastroduodenoscopy showing actively oozing duodenal ulcer in the duodenal bulb.

Our first patient is a 44-year-old male who presented to the emergency department (ED) with the complaint of dizziness, headache, and coffee ground emesis for the past five days. His past medical history was significant for end-stage renal disease requiring hemodialysis, hypertension (HTN), diabetes mellitus (DM), history of hemorrhagic stroke with residual weakness, chronic obstructive pulmonary disease (COPD), and history of Mallory-Weiss tear. In the ED, he was found to have a drop in hemoglobin (Hb) and he was initially medically managed with intravenous fluids, a proton-pump inhibitor, and a blood transfusion. EGD was done, which showed erythematous mucosa in the gastric antrum and one actively oozing crated duodenal ulcer in the duodenal bulb (Forrest Class 1b). The area was injected with epinephrine and coagulation was achieved by using a bipolar probe. This has been presented in Figure 1.

**Figure 2:**
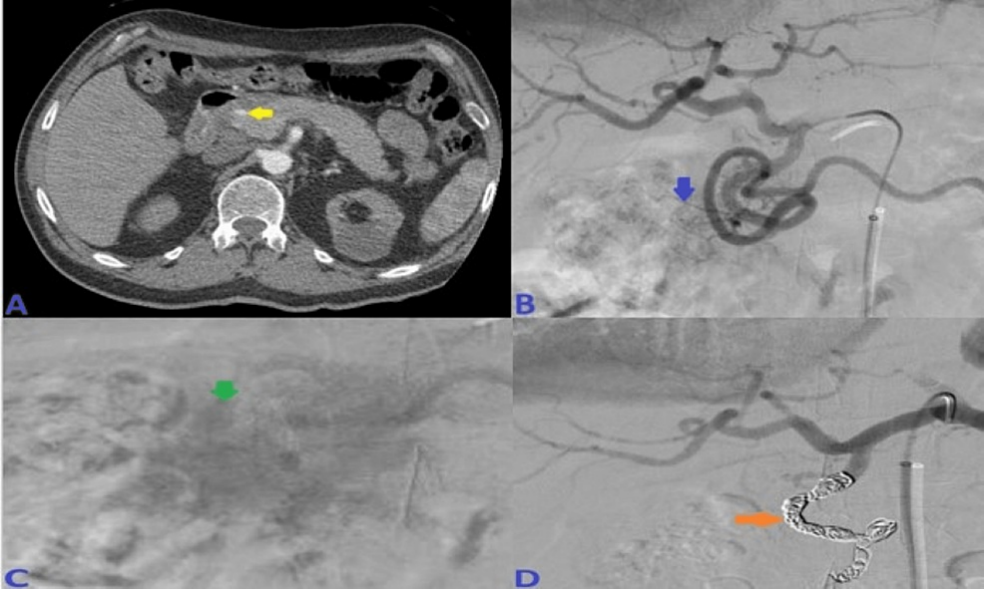
A: CT angiogram axial image of the abdomen demonstrated irregularly dilated and exposed mucosal duodenal arterioles corresponding to actively oozing crated duodenal ulcer in the duodenal bulb seen on prior endoscopy. B: Sequential selective vascular catheterization of celiac and GDA angiograms demonstrated duodenal bleeding territory with many irregularly dilated arteriolar feeders from branches of the GDA with focal blush as seen on delayed venous phase (image C). D: Post-embolization celiac angiogram demonstrated no evidence of active bleeding signs, and complete resolution of the above findings after 13 coils' deployment in “sandwiching” appropriate positions, beyond the takeoff of main GDA, with adequate hepatic arterial inflow at the end of the embolization. CT: computed tomography; GDA: gastroduodenal artery.

His Hb continued to drop without any signs of overt bleeding. Abdominal computed tomography angiography (CTA) demonstrated irregularly dilated and exposed mucosal arterioles along the duodenal lumen. Subsequently, an abdominal aortogram and then sequential selective vascular catheterization of celiac and gastroduodenal artery (GDA) angiograms demonstrated duodenal bleeding territory with many irregularly dilated arteriolar feeders from branches of the GDA. He underwent coil embolization of the GDA using many coils. Post-embolization selective and super-selective angiograms demonstrated no evidence of active bleeding signs, and complete resolution of the above findings after coils' deployment in “sandwiching” appropriate positions, beyond the takeoff of main GDA, with adequate hepatic arterial inflow at the end of the embolization. This has been presented in Figure 2.

There were no further episodes of UGIB and no further drop in Hb, and he was later discharged with outpatient follow-up.

Case 2 

**Figure 3:**
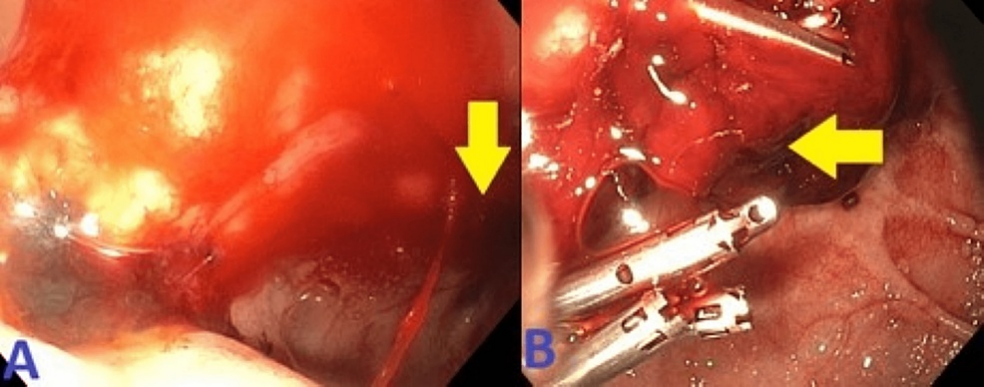
A: Esophagogastroduodenoscopy showing spurting duodenal ulcer. B: Repeat esophagogastroduodenoscopy showing oozing duodenal ulcer.

Our second patient is a 67-year-old male who presented to the ED with the complaint of suprapubic dysuria, black tarry stools, and hematuria for the past three days. His past medical history was significant for HTN, benign prostate hyperplasia, and untreated hepatitis C. In the ED, he was found to have a drop of Hb from his baseline of 9.4 g/dL to 5.8 g/dL. After initial medical management, he underwent EGD, which showed one spurting duodenal ulcer with spurting hemorrhage (Forrest Class Ia). This has been shown in Figure 3A. The ulcer was clipped and injected with epinephrine for hemostasis. Ten days after the EGD he had another episode of UGIB associated with a significant drop in Hb. He underwent a repeat EGD, which showed one nonbleeding duodenal ulcer and one oozing duodenal ulcer with an adherent clot and multiple clips. The area was injected with epinephrine. This has been presented in Figure 3B.

**Figure 4:**
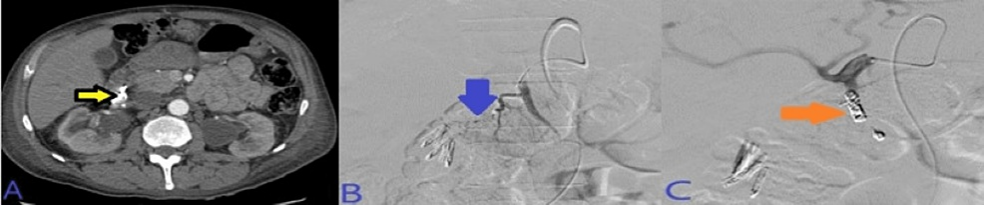
A: CT angiogram of the abdomen demonstrated hyperdense materials/blood products within the second portion of the duodenum adjacent to multiple endoscopic clips. B: Super-selective GDA angiograms using microcatheter systems demonstrated duodenal culprit bleeding with many irregular tortuous arteriolar feeders from superior anterior duodenal arterial branches of the GDA, uncontrolled by adjacent clips placed earlier by endoscopy. C: Post-embolization selective common hepatic angiograms demonstrated no evidence of active bleeding signs, and complete resolution of the above findings after five coils' deployment in “sandwiching” appropriate positions, beyond the takeoff of main GDA and through the proximal aspect of right gastroepiploic artery, with adequate hepatic arterial inflow. CT: computed tomography; GDA: gastroduodenal artery.

His Hb continued to drop after the second EGD and he underwent abdominal CTA, which demonstrated hyperdense materials and blood products within the second portion of the duodenum adjacent to multiple endoscopic clips. Super-selective GDA angiograms using microcatheter systems demonstrated duodenal culprit bleeding with many irregular tortuous arteriolar feeders from superior anterior duodenal arterial branches of the GDA, uncontrolled by adjacent clips placed earlier by endoscopy. Post-embolization selective common hepatic angiograms demonstrated no evidence of active bleeding signs, and complete resolution of the above findings after five coils' deployment in “sandwiching” appropriate positions, beyond the takeoff of main GDA. This has been presented in Figure 4.

His Hb stabilized, and he did not have any further episodes of UGIB.

Case 3 

**Figure 5:**
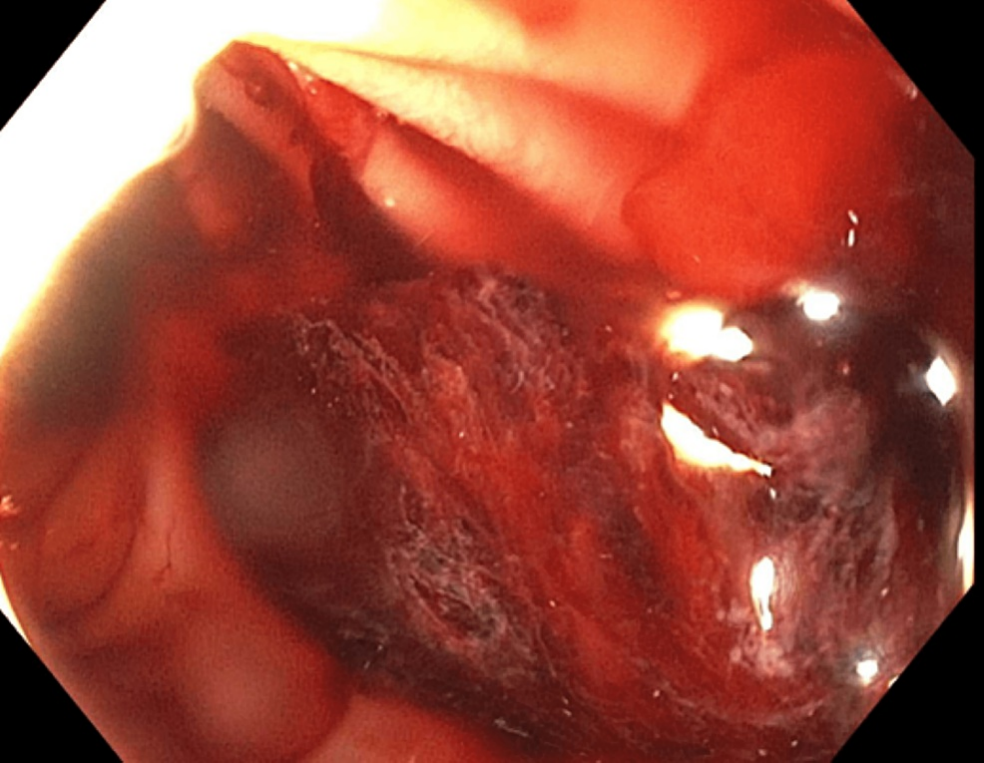
Esophagogastroduodenoscopy showing large amount of bleeding in the second part of the duodenum.

Our third case is a 73-year-old male who presented to the ED with the complaint of hematochezia, dizziness, and watery diarrhea for the past one day. His medication history was significant for DM, HTN, and stage 4 adenocarcinoma of the duodenum, which had metastasized to the liver. After initial medical management, he underwent EGD, which showed a large amount of fresh blood and blood clots in the gastric body and a large villous, fungating mass with bleeding in the second part of the duodenum. No endoscopic therapy could be done due to the mass being very friable. This has been shown in Figure 5.

**Figure 6:**
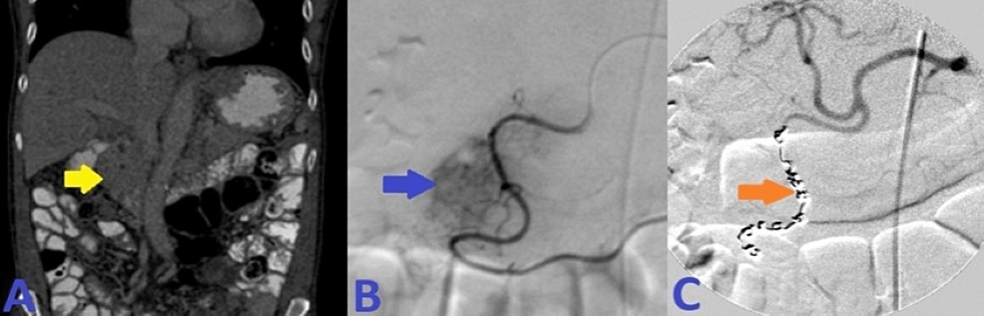
A: CT coronal image demonstrated duodenal fungating mass. B: Super-selective GDA angiograms using microcatheter System demonstrated many pseudoaneurysmal arteriolar tumoral feeders from the GDA branches. C: Post-embolization vascular catheterization for celiac angiogram demonstrated no evidence of active bleeding signs, and complete resolution of the above findings after coils' deployment in “sandwiching” appropriate positions, beyond the takeoff of main GDA, with adequate hepatic arterial inflow at the end of the embolization. CT: computed tomography; GDA: gastroduodenal artery.

The patient underwent a CT abdomen, which showed a duodenal fungating mass. Super-selective GDA angiograms using microcatheter system demonstrated many pseudoaneurysmal arteriolar tumoral feeders from the GDA branches. Post-embolization vascular catheterization for celiac angiogram demonstrated no evidence of active bleeding signs, and complete resolution of the above findings after five coils' deployment. This has been presented in Figure 6.

He was discharged to the nursing home with outpatient follow-up.

Case 4 

**Figure 7:**
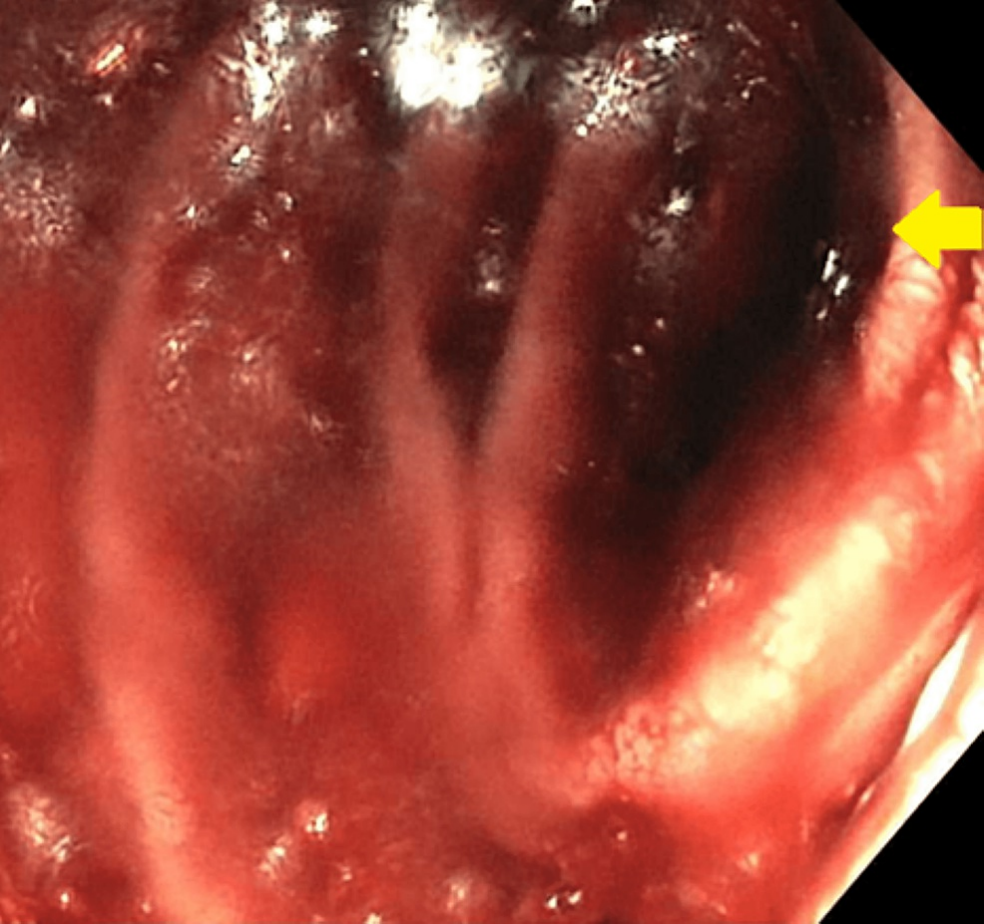
Small bowel enteroscopy showing diffuse mucosal oozing in the second portion of the duodenum.

Our fourth case is a 64-year-old woman who presented to the ED with a complaint of hematochezia for the past 10 days. Her past medical history was significant for coronary artery disease, HTN, atrial fibrillation, and rheumatic heart disease. EGD showed a small blood clot in the first portion of the duodenum. Her Hb continued to drop, so a small bowel enteroscopy was done which showed diffuse mucosal oozing and multiple ulcers in the second portion of the duodenum (Forrest Class IIa). This has been shown in Figure 7.

**Figure 8:**
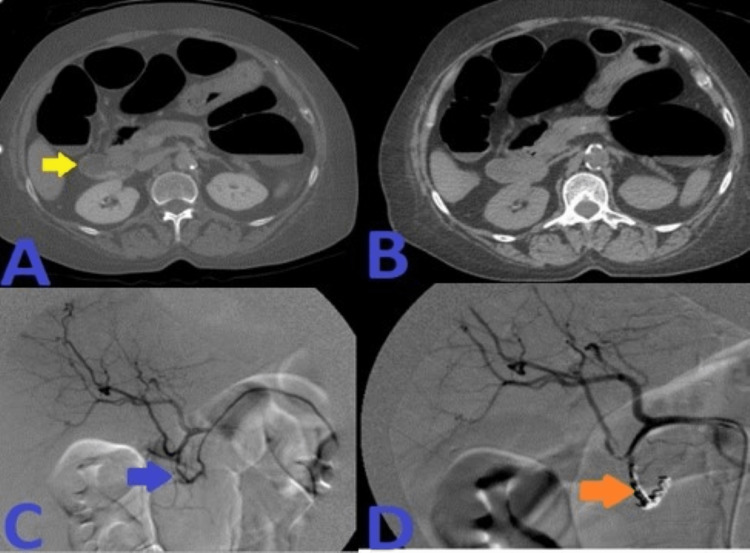
A: CT angiogram abdomen with contrast showed hyperdense material seen on the portal venous phase within the second portion of the duodenum, not present on the noncontrast phase of the CT as seen in image B. C: Super-selective GDA angiograms using microcatheter system demonstrated duodenal culprit bleeding from lateral superior branch of the GDA. D: Post-embolization selective and super-selective angiograms demonstrated no evidence of active bleeding signs, and complete resolution of the above findings with four deployed coils in appropriate positions, just beyond the takeoff of an accessory right hepatic artery coming off as anatomical variant from the GDA. CT: computed tomography; GDA: gastroduodenal artery.

Abdominal CTA was done which showed hyperdense material seen on the portal venous phase within the second portion of the duodenum. Super-selective GDA angiograms using microcatheter System demonstrated duodenal culprit bleeding from a lateral superior branch of the GDA. Post-embolization selective and super-selective angiograms demonstrated no evidence of active bleeding signs, and complete resolution of the above findings with four deployed coils in appropriate positions. This has been presented in Figure 8.

Her further hospital course was complicated by respiratory failure requiring tracheostomy and eventually percutaneous endoscopic gastrostomy (PEG) placement. She was discharged to a skilled nursing facility.

Case 5 

**Figure 9:**
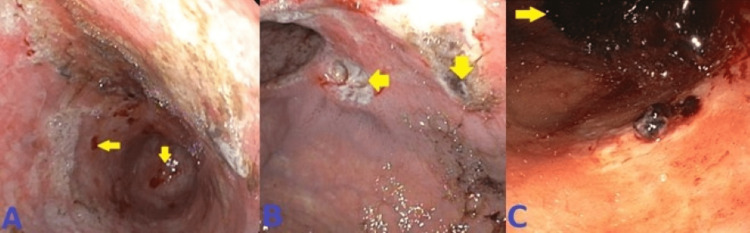
A and B: Esophagogastroduodenoscopy showing nonbleeding cratered gastric ulcer with a visible artery in gastric body and antrum. C: Showing large amount of blood in the gastric fundus and antrum.

Our fifth case is an 84-year-old man who was sent from a nursing home to the ED due to an episode of hematemesis. His medical history was significant for mild dementia, atrial fibrillation (was not on anticoagulation), and metastatic prostate cancer. He had a significant drop in Hb from his baseline of 13.7 g/dL to 7.7 g/dL. EGD was done, which showed diffusely erythematous mucosa in the gastric antrum along with a few nonbleeding cratered gastric ulcer with a visible artery in the gastric body and antrum (Forrest Class IIa). It was treated with bipolar cautery. This has been shown in Figure 9A, 9B. Hb value continued to drop, which required multiple blood transfusions. A repeat EGD was done, which showed a large amount of blood in the gastric fundus and antrum precluding visualization. A nonbleeding superficial gastric ulcer with no stigmata of bleeding was found in the gastric antrum. There was one nonbleeding cratered duodenal ulcer with no stigmata of bleeding in the duodenum's third portion. This has been shown in Figure 9C.

**Figure 10:**
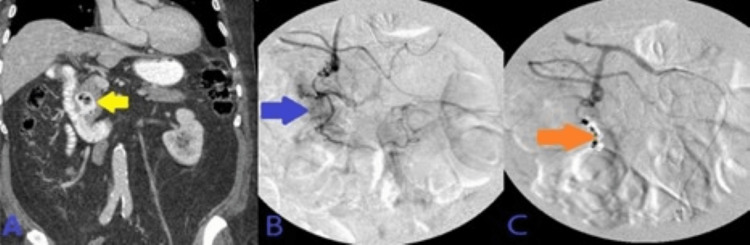
A: CT demonstrated duodenal outpouching filled with oral contrast correlating with duodenal ulceration seen on endoscopy. B: Super-selective GDA arterial and venous angiograms using microcatheter System demonstrated many pseudoaneurysmal duodenal culprit bleeding territories from IPDA coming off the GDA. C: Post-embolization vascular catheterization for celiac angiogram demonstrated no evidence of active bleeding signs, and complete resolution of the above findings after three coils' deployment along the IPDA in appropriate positions, beyond the takeoff of gastroepiploic artery, with adequate hepatic arterial inflow at the end of the embolization. CT: computed tomography; GDA: gastroduodenal artery; IPDA: inferior pancreaticoduodenal artery.

Super-selective GDA arterial and venous angiograms showed many pseudoaneurysmal duodenal culprit bleeding territories from an inferior pancreaticoduodenal artery (IPDA) coming off the GDA. Embolization was done with three coils' deployment along the IPDA. This has been presented in Figure 10.

His hospital course was complicated by respiratory failure requiring tracheostomy and PEG. In view of his poor clinical status and prognosis, the family opted for no cardiopulmonary resuscitation in case of any cardiopulmonary arrest. Later he developed asystole and expired.

Case 6 

**Figure 11:**
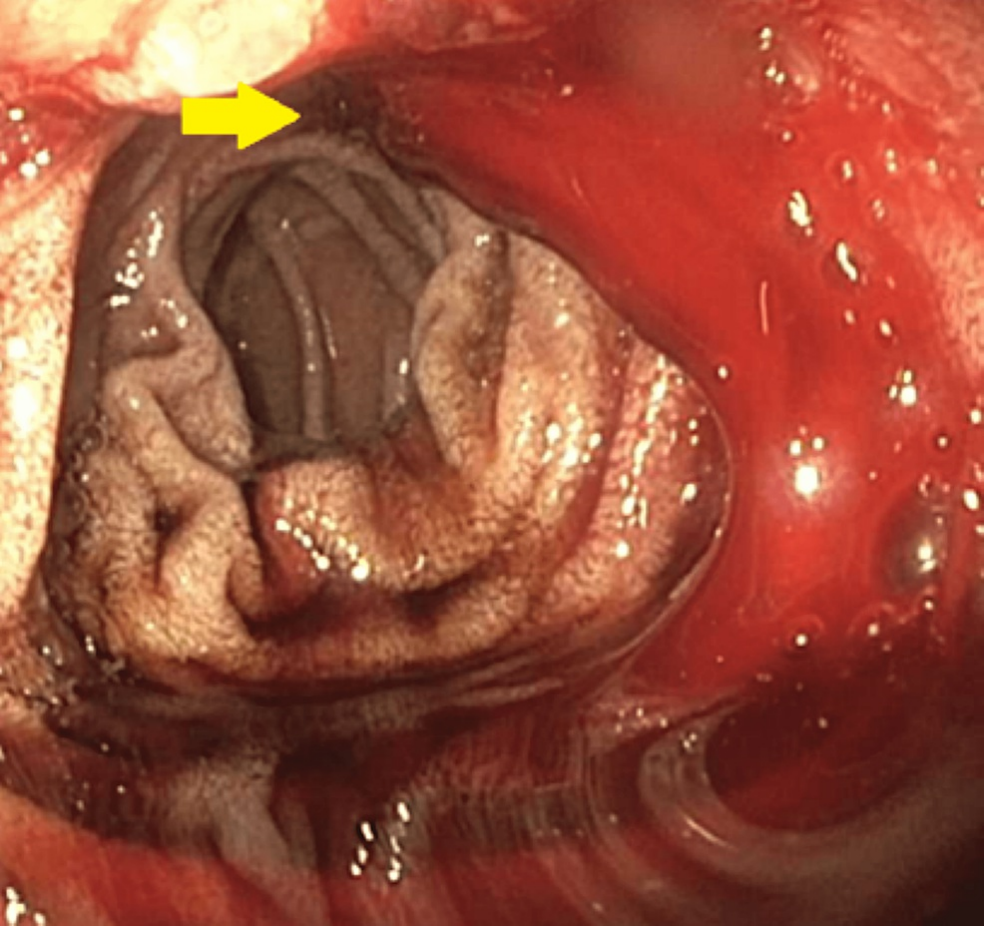
Esophagogastroduodenoscopy showing oozing duodenal ulcer.

Our sixth case is a 65-year-old male who was sent from a nursing home to the ED for shortness of breath. His medical history was significant for acquired immunodeficiency syndrome (AIDS), dementia, and COPD. He was admitted for hypoxic respiratory failure due to pneumonia. His hospital course was complicated with an episode of melena and a 4 g/dL drop in Hb. CT abdomen showed a hyperdense material seen within the second portion of the duodenum. EGD showed a few nonbleeding duodenal ulcers and one oozing duodenal ulcer (Forrest Ib). The area was injected with epinephrine and cautery was done with a monopolar probe. This has been shown in Figure 11.

**Figure 12:**
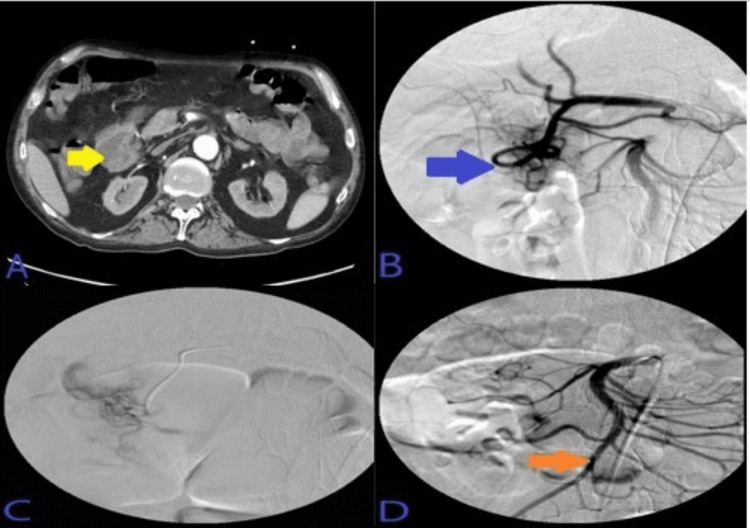
A: CT angiogram of the abdomen showed hyperdense material seen within the second portion of the duodenum compatible with oozing blood seen on endoscopy. B: Selective celiac angiograms demonstrated irregularly tortuous duodenal arterioles as the culprit of bleeding from branch of the GDA with hypertrophic IPDA shunting toward the SMA, therefore no coils were used to avoid non-target migration and bowel ischemia. C: Super-selective GDA angiograms using microcatheter system for embolization using particulate polyvinyl alcohol (PVA) 500 micron particle with full stasis of flow. D: Post-embolization selective SMA angiogram demonstrated patent jejunal, IPDA and ileocecal and colic arteries with no signs of backflow duodenal bleeding. CT: computed tomography; IPDA: inferior pancreaticoduodenal artery; SMA: superior mesenteric artery; GDA: gastroduodenal artery.

The patient had another episode of bleeding. Selective celiac angiograms demonstrated irregularly tortuous duodenal arterioles as the culprit of bleeding from a branch of the GDA with hypertrophic IPDA shunting toward the superior mesenteric artery (SMA). Embolization was done using particulate PVA 500 micron particle. This has been shown in Figure 12.

He had no further episode of gastrointestinal bleeding post embolization. His respiratory failure continue to worsen. He developed asystole not responding to resuscitative efforts and passed away.

Case 7

**Figure 13:**
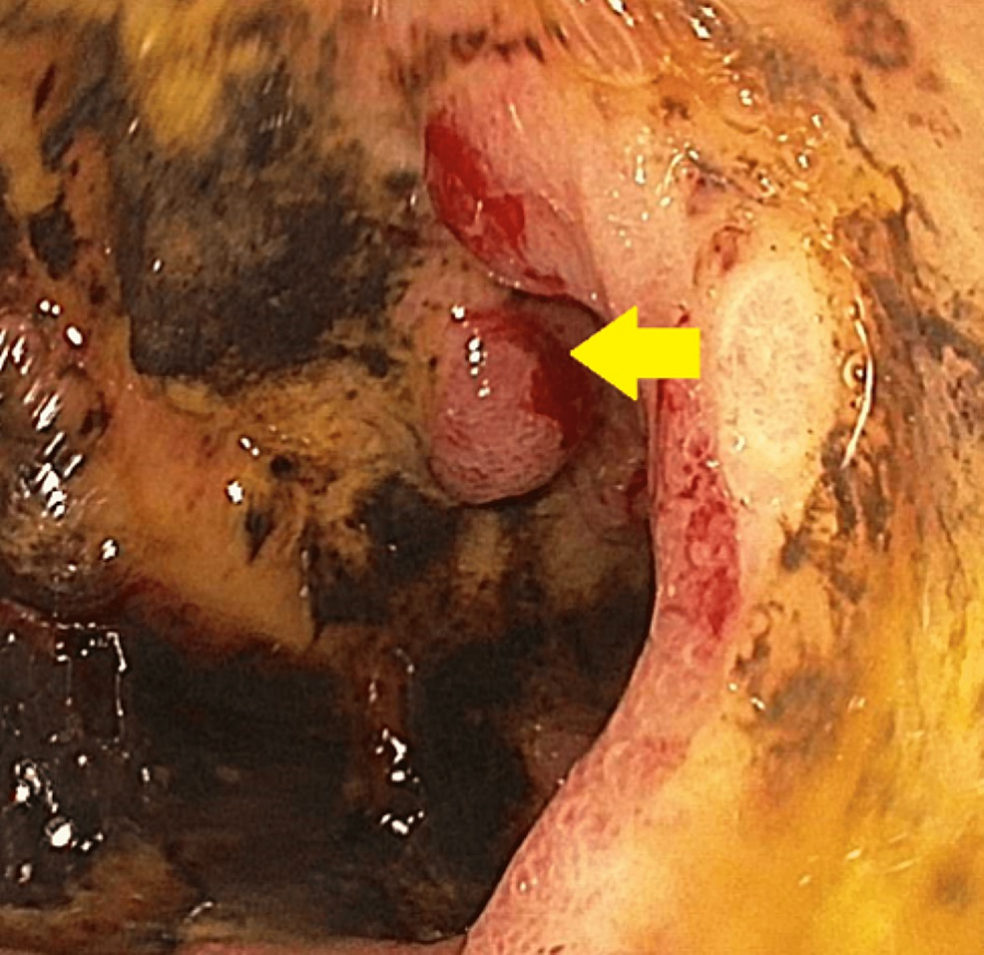
Esophagogastroduodenoscopy showing oozing duodenal ulcer in the duodenal bulb.

Our seventh case is a 55-year-old male who was sent to the ED after being found unresponsive at his assisted living facility. His medical history was significant for DM, HTN, chronic kidney disease, and polysubstance abuse. He was admitted for hypoxic hypercapnic respiratory failure due to drug overdose and aspiration pneumonia. The hospital course was complicated with an episode of coffee ground emesis. Computed tomography (CT) of the abdomen demonstrated dense-fluid-filled duodenal wall thickening. EGD showed multiple dispersed nonbleeding erosions with no stigmata of recent bleeding, and one oozing cratered 30 mm duodenal ulcer (Forrest Class IB) in the duodenal bulb. The area was injected with epinephrine. Thermal therapy was deferred due to the risk of perforation. This has been shown in Figure 13.

**Figure 14:**
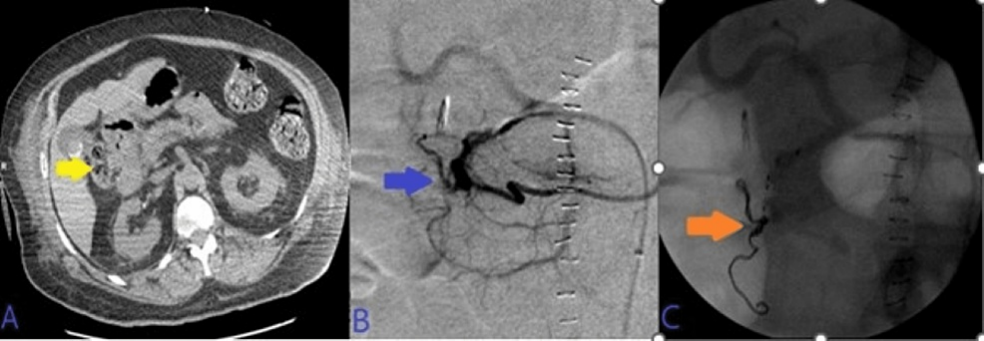
A: CT of the abdomen demonstrated dense-fluid-filled duodenal wall thickening and ulceration crater as seen on endoscopy. B: Super-selective GDA angiograms demonstrated many focal bleeding blush adjacent to endoscopic clip. C: Post-embolization celiac angiogram demonstrated no evidence of active bleeding signs, and complete resolution of the above findings after deployment of one coil and two vascular plugs in appropriate positions along the GDA. CT: computed tomography; GDA: gastroduodenal artery.

He had another episode of coffee ground emesis with a significant drop in Hb. EGD was repeated, which shows single small erosion in the gastric antrum and one nonbleeding cratered 30 mm duodenal ulcer in the duodenal bulb. The area was treated again with epinephrine. Despite two attempts at endoscopic therapy, he continued to have a drop in Hb requiring multiple blood transfusions. CT of the abdomen was done, which showed dense-fluid-filled duodenal wall thickening . He underwent exploratory laparotomy and EGD by the surgical team. He was found to have inflammatory thickening of the duodenal bulb and the second portion of the duodenum, a large ulcer in the duodenal bulb, which was very close to the ampulla and three more ulcers in the distal duodenum. All the ulcers were covered with a firm clot with no sign of active bleeding. The options of distal gastrectomy, duodenotomy, and vagotomy were not rendered feasible as it would have required drainage via enterotomy and a high possibility of an anastomotic leak. Later during the course, he had two episodes of hematochezia with a drop in Hb. Another EGD was done, and he was found to have one nonbleeding cratered duodenal ulcer with a nonbleeding visible vessel (Forrest Class IIa) in the duodenal bulb extending into the second part of the duodenum. It was treated with bipolar cautery, argon plasma coagulation, and a clip was placed. Despite multiple attempts at endoscopic therapy, he continued to bleed and eventually embolization with the deployment of one coil and two vascular plugs was done. This has been presented in Figure 14.

He had no further episodes of bleeding and was discharged to a skilled nursing facility.

Case 8

**Figure 15:**
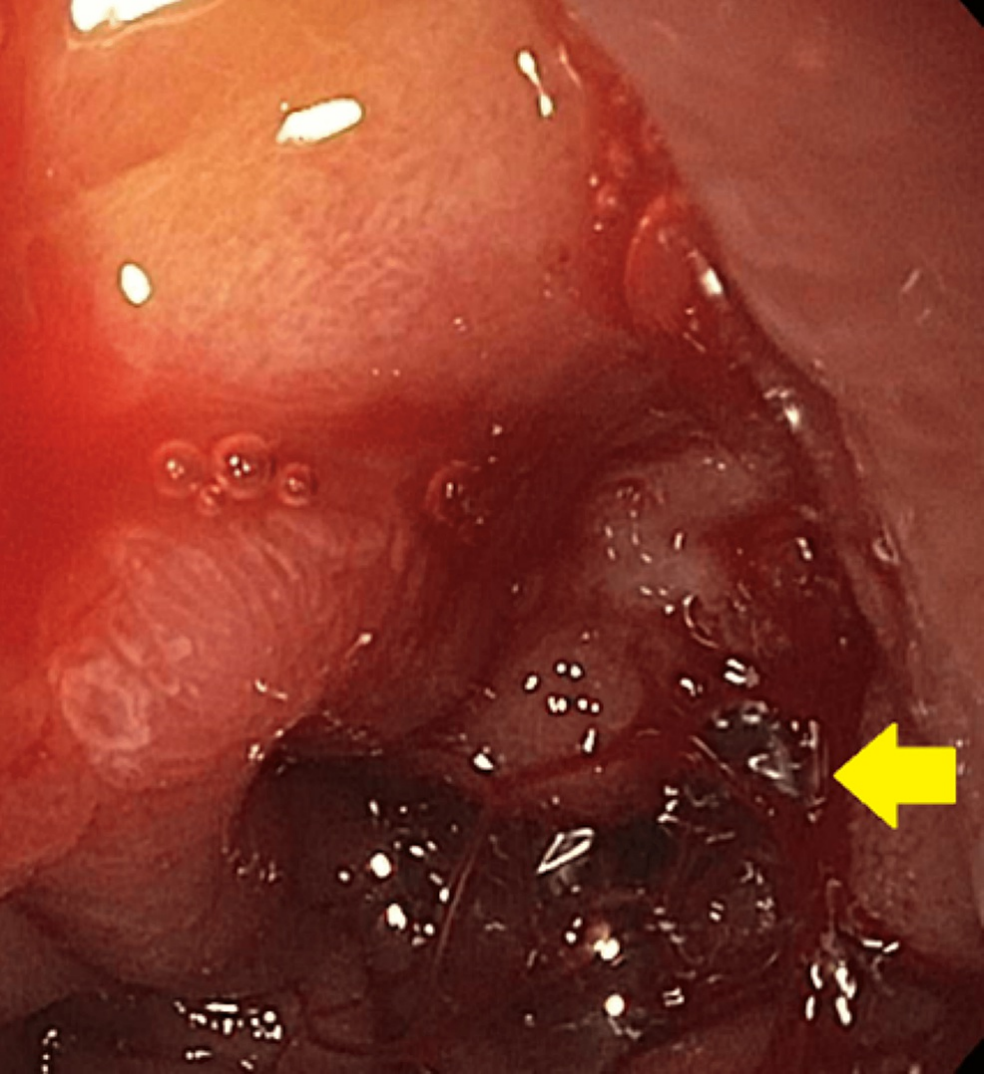
Esophagogastroduodenoscopy showing actively bleeding ulcer in the second part of the duodenum.

Our eighth case is a 53-year-old male who was sent to the ED from a nursing home for melena. His medical history was significant for hepatitis C, cirrhosis (Model for End-Stage Liver Disease MELD score 14), ventilator dependence, and history of pulmonary embolism, HTN, and DM. After initial medical management, the patient underwent EGD, which showed three ulcers in the second part of the duodenum. Active bleeding and blood clots were seen in the duodenum. The first ulcer, which was approximately 10 mm in size, was actively bleeding. Three hemostatic clips were placed for hemostasis (Forrest Ib). The second ulcer was approximately 8 mm and was noted to have mild oozing, which stopped spontaneously. The third ulcer had a clean base and was approximately 10 mm in size. This has been shown in Figure 15.

**Figure 16:**
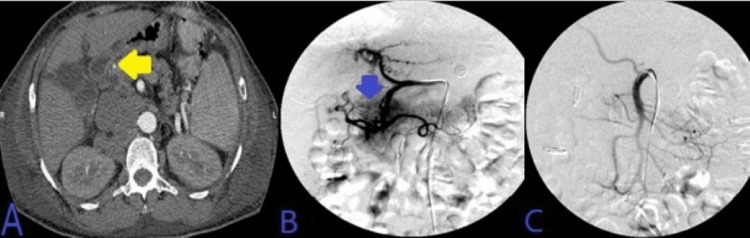
A: CT scan with contrast of the abdomen showed hypertrophic submucosal arterioles along the second portion of the duodenum compatible with oozing seen on endoscopy. B: Selective celiac angiograms demonstrated irregularly tortuous duodenal arterioles as the culprit of bleeding from branch of the GDA with shunting toward the SMA, therefore no coils were used at this time to avoid non-target migration and bowel ischemia. C: Super-selective GDA angiograms with embolization using particulate polyvinyl alcohol (PVA) 500 was done. Post-embolization selective celiac and SMA angiograms demonstrated patent jejunal and ileocecal and colic arteries with replaced right hepatic artery with no signs of backflow duodenal blushes or bleeding. CT: computed tomography; GDA: gastroduodenal artery; SMA: superior mesenteric artery.

Selective celiac angiograms demonstrated irregularly tortuous duodenal arterioles as the culprit of bleeding from a branch of the GDA with shunting toward the SMA. Embolization using particulate PVA 500 micron particle was done. He had no further bleeding episodes post embolization. This has been presented in Figure 16.

His hospital course was complicated with severe sepsis with septic shock secondary to multifocal pneumonia. His clinical condition continued to deteriorate. His family opted for no cardiopulmonary resuscitation in case of any cardiac arrest, and he eventually expired.

Case 9

**Figure 17:**
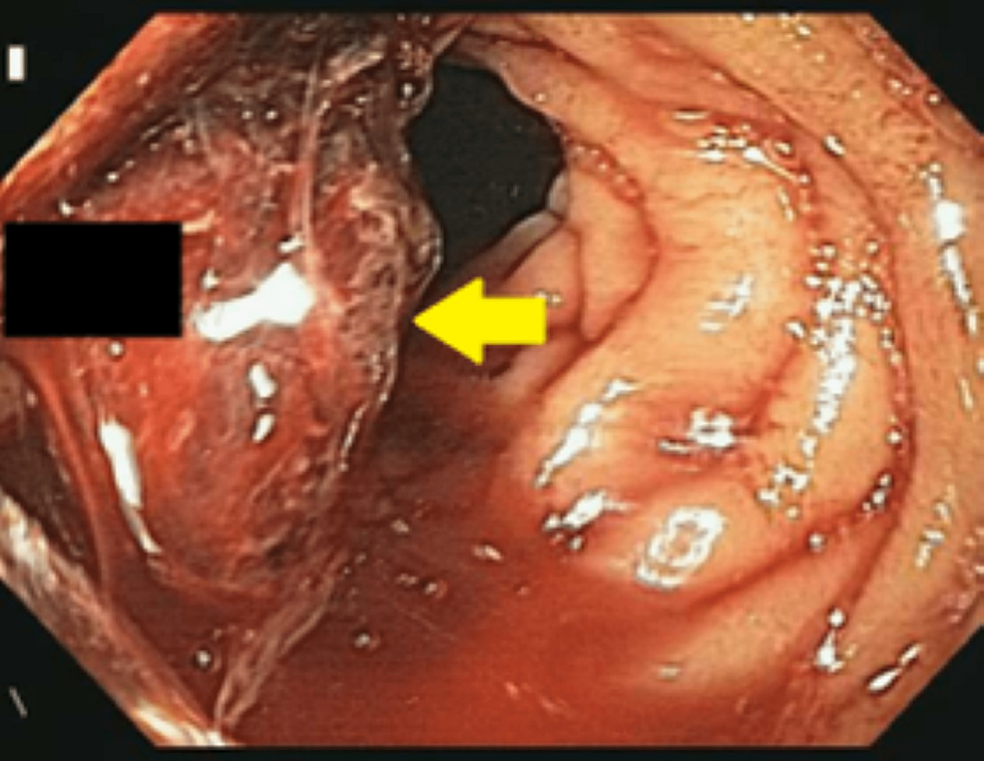
Esophagogastroduodenoscopy showing large friable necrotic malignant-appearing infiltrative mass in the second part of the duodenum.

Our ninth case is a 67-year-old man who was brought to the ED for shock and hypotension. His family reported jaundice and multiple episodes of hematemesis for the last one week. His medical history was significant for hypertension, diabetes, and history of cerebrovascular accidents. He was intubated and underwent endoscopic evaluation. EGD showed a large friable necrotic, malignant-appearing infiltrative mass with large adherent blood clots and stigmata of recent bleeding in the second part of the duodenum. This has been shown in Figure 17.

**Figure 18:**
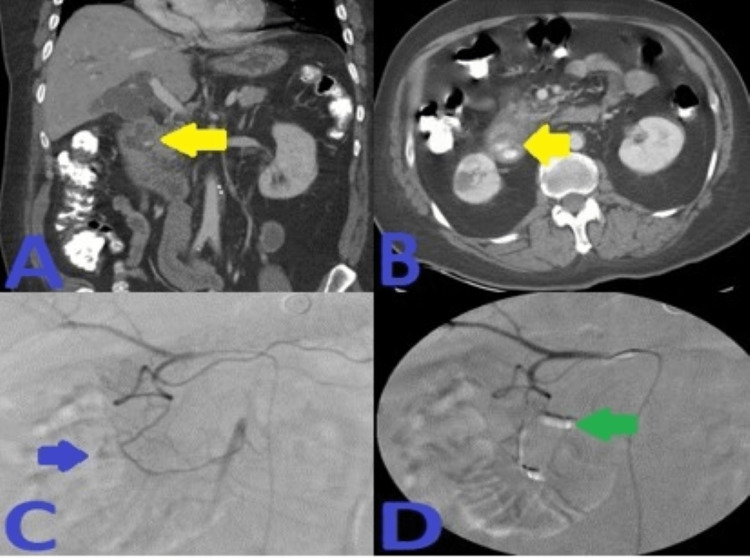
A: CT axial arterial phase demonstrated infiltrative mass with active bleeding in the second part of the duodenum with layering of contrast in the dependent aspect of the duodenum on venous phase as seen on image B. C: Super-selective GDA angiograms using microcatheter system demonstrated active duodenal bleeding from IPDA coming off the GDA with contrast flowing freely and actively from the GDA branches to the duodenum, as seen on CT scan. D: Post-embolization celiac angiogram demonstrated no evidence of active bleeding signs, and complete resolution of the above findings after coils deployment along IPDA in “sandwiching” appropriate positions, beyond the takeoff of gastroepiploic artery, with adequate hepatic arterial flow. CT: computed tomography; IPDA: inferior pancreaticoduodenal artery; GDA: gastroduodenal artery.

Abdomen computed tomography angiogram (CTA) demonstrated infiltrative mass with active bleeding in the second part of the duodenum. Embolization using coils along IPDA was done. This has been presented in Figure 18.

There were no further episodes of gastrointestinal bleeding. He underwent endoscopic retrograde cholangiopancreatography (ERCP) for obstructive jaundice and biliary dilatation. Endoscopic ultrasound (EUS) was done, which showed diffuse nodular mucosa in the duodenal bulb and a hypoechoic 2 x 2 cm lesion in the pancreatic head. Later on, he underwent another EUS with the fine-needle aspiration of the hypoechoic lesion in the pancreatic head. Histopathology revealed the mass to be invasive moderately differentiated adenocarcinoma. He was referred to oncology and surgery for further management.

Case 10 

**Figure 19:**
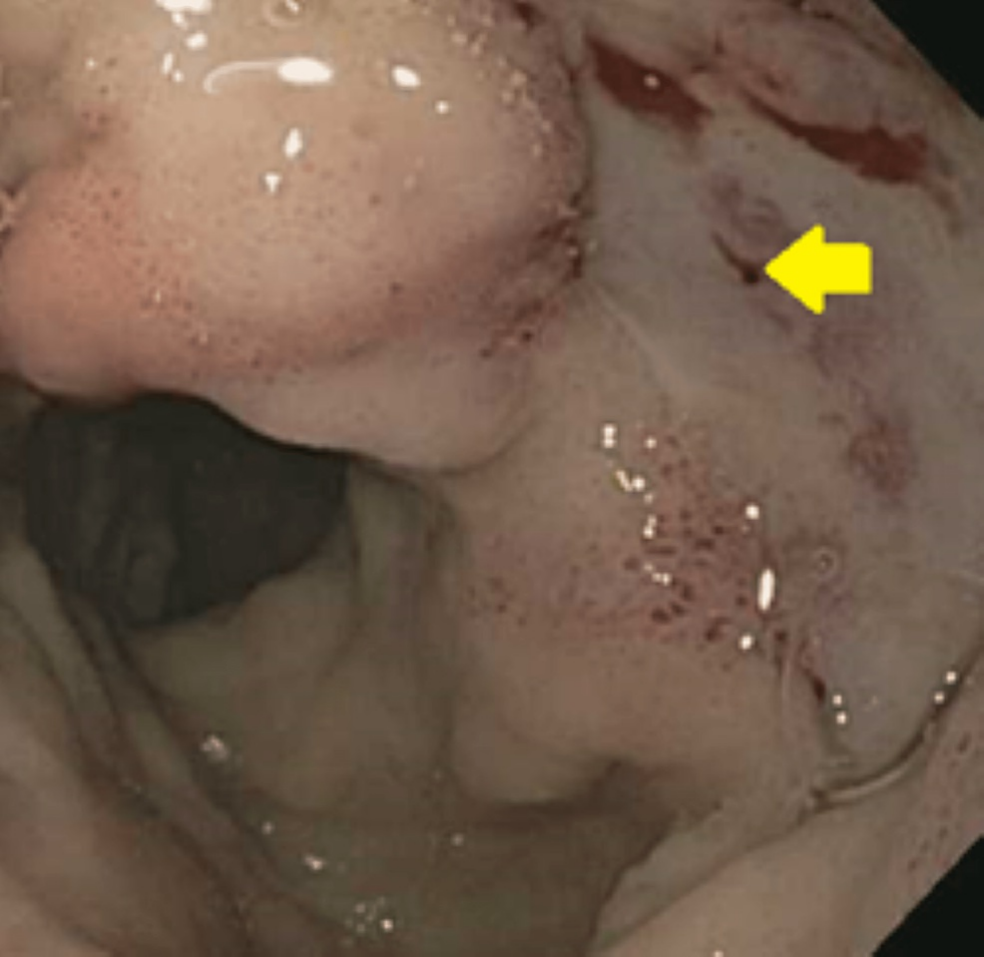
Esophagogastroduodenoscopy showing one bleeding cratered duodenal ulcer with a visible vessel in the duodenal bulb.

Our tenth case is of a 59-year-old man who presented to the ED with a complaint of hematochezia for the past one day. His medical history was significant for hemorrhagic cerebrovascular accident history, high-grade neuroendocrine tumor of the lung with extensive metastasis, and alcoholic liver cirrhosis. EGD was done, which showed one bleeding cratered duodenal ulcer with a visible vessel in the duodenal bulb. The area was injected with epinephrine and electrocautery was done. This has been shown in Figure 19.

**Figure 20:**
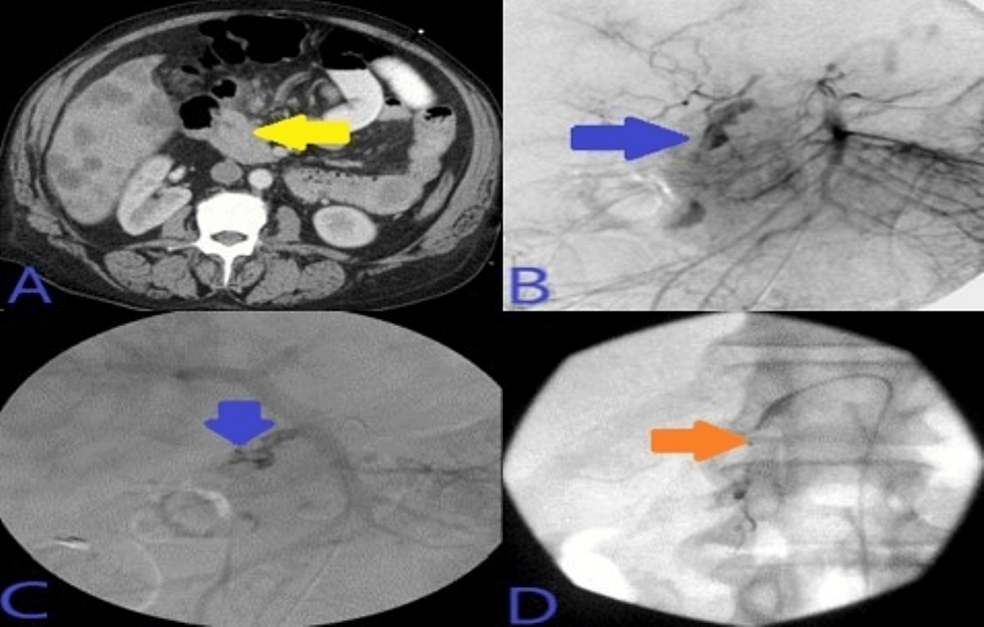
A: CT demonstrated hypertrophic submucosal arterioles along the duodenum compatible with oozing ulceration as seen on endoscopy. B: SMA angiogram demonstrating large active and delayed portal venous phase (image C). D: Post-embolization celiac angiogram demonstrated no evidence of active bleeding signs, and complete resolution of the above findings after deployment of two coils and one vascular plug in appropriate positions along the GDA. CT: computed tomography; SMA: superior mesenteric artery; GDA: gastroduodenal artery.

He continued to have hematochezia. A colonoscopy was done, which showed one 6 mm polyp in the descending colon and blood in the sigmoid colon and descending colon. A repeat EGD showed one cratered duodenal ulcer with spurting hemorrhage (Forrest Class Ia) in the duodenal bulb. Two clips were placed to stop the bleeding as coagulation using a bipolar probe was unsuccessful. Three days after the EGD the patient had another episode of rectal bleeding. Abdominal CTA demonstrated hypertrophic submucosal arterioles along the duodenum. Embolization was done using two coils and one vascular plug in appropriate positions along the GDA. This has been presented in Figure 20.

He had no further episodes of bleeding. His hospital course was complicated by lung collapse and bronchoscopy showed a fungating mass occluding the lingular aperture. His family opted for no resuscitation and wanted palliative treatment. The patient later expired.

A brief summary of the endoscopic and Interventional Radiology (IR)-guided intervention done for all the patients has been presented in Tables 1, 2. 

**Table 1:** Demographics, EGD, and CT findings, EGD and interventional radiology intervention, and clinical outcome of cases 1 to 5 EGD: esophagogastroduodenoscopy; CT: computed tomography; GDA: gastroduodenal artery; IPDA: inferior pancreaticoduodena artery.

	Case 1	Case 2	Case 3	Case 4	Case 5
Age (years)	44	67	73	64	84
Gender	Male	Male	Male	Female	Male
Presenting complaint	Hematemesis	Melena	Hematochezia	Hematochezia	Hematemesis
EGD findings	One actively oozing duodenal ulcer (Forrest Ib)	One spurting duodenal ulcer with spurting hemorrhage (Forrest Ia)	Large villous, fungating and ulcerative mass	No active bleeding lesion however with adherent clot in the first portion of duodenum (Forrest IIa). Enteroscopy with mucosal oozing from the second part of duodenum	Few nonbleeding gastric ulcers (Forrest IIa)
Endoscopic intervention	Epinephrine injection and electrocautery	Epinephrine Injection and clips placed	No therapeutic management done as the mass was very friable	None	Electrocautery
CT abdomen findings	Irregularly dilated and exposed mucosal duodenal arterioles	Hyperdense materials/blood products within the second portion of the duodenum adjacent to multiple endoscopic clips	Duodenal fungating mass	Hyperdense material seen on the portal venous phase within the second portion of the duodenum	Duodenal outpouching filled with oral contrast
Angiogram findings	Duodenal bleeding territory with many irregularly dilated arteriolar feeders from the branch of gastroduodenal artery (GDA)	Duodenal culprit bleeding with many irregular tortuous arteriolar feeders from superior anterior duodenal arterial branches of the GDA	Pseudoaneurysmal arteriolar tumoral feeders from the GDA branches	Duodenal culprit bleeding from lateral superior branch of the GDA	Many pseudoaneurysmal duodenal culprit bleeding territory from Inferior pancreaticoduodena artery (IPDA) coming off the GDA
Interventional radiology intervention	13 coils deployed beyond the takeoff of main GDA	5 coils deployed beyond the takeoff of main GDA	5 coils deployed beyond the takeoff of main GDA	4 coils deployed just beyond the takeoff of an accessory right hepatic artery coming off as anatomical variant from the GDA	3 coils deployed along the IPDA beyond the takeoff of gastroepiploic artery
Outcome	Alive with no further known duodenal bleeding episodes after angioembolization	Alive with no further known duodenal bleeding episodes after angioembolization	Alive with no further known duodenal bleeding episodes after angioembolization	Alive with no further known duodenal bleeding episodes after angioembolization	Expired - likely due to severe septic shock from pneumonia with multidrug-resistant *Escherichia coli*

**Table 2:** Demographics, EGD and CT findings, EGD and interventional radiology intervention, and clinical outcome of cases 6 to 10 CT: computed tomography; GDA: gastroduodenal artery; IPDA: inferior pancreaticoduodenal artery; SMA: superior mesenteric artery.

	Case 6	Case 7	Case 8	Case 9	Case 10
Age (years)	65	55	53	67	59
Gender	Male	Male	Male	Male	Male
Presenting complaint	Melena	Hematemesis	Melena	Hematemesis	Hematochezia
EGD findings	Few nonbleeding duodenal ulcers and one oozing duodenal ulcer with a visible vessel ( Forrest Ib)	Multiple dispersed nonbleeding erosions with no stigmata of recent bleeding, and one oozing cratered 30 mm duodenal ulcer (Forrest Ib)	One bleeding duodenal ulcer, one oozing duodenal ulcer (Forrest Ib) and one nonbleeding duodenal ulcer	Large friable necrotic, malignant-looking infiltrative bleeding mass in duodenum	One bleeding cratered duodenal ulcer with a visible vessel in the duodenal bulb (Forrest IIa)
EGD intervention	Epinephrine injection and electrocautery	Epinephrine injection. Thermal therapy deferred due to risk of perforation	Clips placed	None	Epinephrine injection and electrocautery
CT abdomen findings	Hyperdense material seen within the second portion of the duodenum	Dense-fluid-filled duodenal wall thickening and ulceration crater	Hypertrophic submucosal arterioles along the second portion of the duodenum	Infiltrative mass with active bleeding in the second part of the duodenum	Hypertrophic submucosal arterioles along the duodenum
Angiogram findings	Irregularly tortuous duodenal arterioles as the culprit of bleeding from branch of the gastroduodenal artery (GDA) with hypertrophic inferior pancreaticoduodena artery (IPDA) shunting toward the superior mesenteric artery (SMA)	Many focal bleeding blush adjacent to endoscopic clip	Irregularly tortuous duodenal arterioles as the culprit of bleeding from branch of the GDA with shunting toward the SMA	Active duodenal bleeding from IPDA coming off GDA	irregular and spastic GDA with questionable focal bleeding blush adjacent to endoscopic clips
Interventional radiology intervention	Particulate polyvinyl alcohol (PVA) 500 micron particle used in the GDA	1 coil and 2 vascular plugs deployed along the GDA	Particulate PVA 500 micron particle used in the GDA	5 coils deployed along IPDA beyond the takeoff of gastroepiploic artery	1 coil and 2 vascular plugs deployed along the GDA
Outcome	Expired - likely due to severe sepsis with septic shock secondary to pneumonia from multidrug-resistant gram-negative organism	Alive with no further known duodenal bleeding episodes after angioembolization	Expired - likely due to severe sepsis with septic shock secondary to multifocal pneumonia	Alive with no further known duodenal bleeding episodes after angioembolization	Expired - likely due to respiratory failure due to left lung collapse secondary to endobronchial lesion

## Discussion

Initial management of UGIB includes fluid resuscitation, proton-pump inhibitors, and transfusion of blood products. Once hemodynamic stability is achieved, endoscopic management with epinephrine, thermal therapy, or clip placement is usually employed based on the Forrest classification of the target lesion in the gastrointestinal tract [5]. JA Forrest et al. first described Forrest classification in 1974 [2]. Forrest classification has been presented in Table 3.

**Table 3:** Forrest classification

Forrest class	Endoscopic findings
Class 1	Acute hemorrhage/active bleeding
1A	Active spurting
1B	Active oozing
Class 2	Sign or stigmata of recent hemorrhage
2A	Nonbleeding visible vessel
2B	Adherent clot
2C	Flat pigmented spot
Class 3	Lesions without active bleeding - clean ulcer base

Conventionally, surgical or radiological endovascular interventions like TAE have been employed for the management of UGIB once endoscopic intervention fails [2]. In our case series review, we focused on the use of TAE in the management of UGIB nonresponsive and not controlled by conventional endoscopic therapies.

Endoscopic treatment, as the gold standard treatment for UGIB, does not always successfully control bleeding or rebleeding in 10-25% of patients. TAE, a less risky alternative to surgical interventions and TAE, can be considered the first-line treatment for massive duodenal bleeding if endoscopic treatment fails in patients with bleeding from duodenal ulcers that cannot be controlled by conservative treatment and immediate endoscopic hemostatic therapy [6,7].

According to American College of Gastroenterology guidelines, endoscopy is recommended within 24 hours of presentation of UGIB and 12 hours if high-risk clinical features are present. Repeat or second-look endoscopy is recommended for recurrent bleeding focusing on achieving hemostasis. Up to 10% of patients may not respond to initial endoscopic therapy [8,9].

After a second look at endoscopy, if bleeding recurs, TAE is generally considered the next step in the management of UGIB. Conservative surgeries such as ulcer suture, ligation of the GDA, and radical surgeries such as distal gastrectomy with partial duodenectomy emergency surgical procedures have been described with high morbidity rate (50%) and mortality rates (30%), when considering patients' ages with a high incidence of comorbid diseases [10]. Several series and meta-analyses subsequently confirmed the role, efficacy, success rate, and safety of TAE in the management of UGIB with superiority of TAE for the length of stay, rebleeding, and complication rates [11-14].

Common indications for endovascular TAE include relapse bleeding after the endoscopic intervention, failure of endoscopic hemostasis, or in the case of an endoscopically nonlocalizable source of bleeding or inaccessible duodenal lumens such as in cases of post gastric bypass or Whipple surgeries. The most common indication for endovascular therapy for our patients in the study happens to be failed endoscopic intervention in controlling UGIB and a minor proportion of the cases were referred for endovascular management because of inaccessibility of the lesion endoscopically. Contraindications for endovascular therapies are usually relative and involve consumption coagulopathy.

All our TAE procedures were performed under local anesthesia by an interventional radiologist with an extensive experience in transcatheter embolization. The Seldinger technique was used with femoral arteriotomy approach via 5 French (Fr) access sheath with selective catheterization of the visceral arteries, mainly the celiac axis, using 5 Fr curved tip followed by super-selective catheterization of the GDA with the assistance of 2.4, 2.8, or 3 Fr microcatheters loaded with microwires of 0.018 inches. After identifying the site of bleeding on angiography and/or targeting the endoscopic clips if not clearly seen on real-time angiography in the cath lab suite, the culprit artery was catheterized and TAE with coils or PVA particles or vascular plug devices was performed until flow stasis or absence of blushes on the completion arteriography for our patients. Other embolic materials such as liquid N-butyl cyanoacrylate glue (NBCA), ethylene-vinyl alcohol copolymers (Onyx), tris-acryl gelatin microspheres, vascular plug devices, and gelatin sponges are used as well to manage UGIB. Intra-arterial infusion of vasopressin or placement of covered stents for vascular injuries is also described among endovascular therapies for refractory UGIB [3].

The success rate of angiographic embolization in recurrent upper GIB refractory to medical and endoscopic therapy is reported to be as high as >90% with a rebleeding rate of 9-47% [15]. EGD can also contribute to guiding the endovascular intervention planning to selectively catheterize the culprit bleeding vessel branch by targeting the endoscopic clips marking if no significant contrast extravasation can be easily detected angiographically. Significant factors contributing to the failure of TAE or rebleeding following the procedure include coagulopathy, delayed time to angiography, use of massive transfusions, previous abdominal surgeries, presence of multi-organ system failure, bleeding related to trauma or invasive procedures, and malignancy-associated bleeding [16,17]. Surgical intervention may be considered for patients with recurrent or refractory UGIB despite adequate attempts at endoscopic therapy. Compared with surgical intervention, patients receiving IR-guided embolization in acute recurrent UGIB have similar or better post-procedural outcomes and minimal complications [18]. None of our 10 patients experienced rebleeding after TAE.

Complications of the TAE in the UGIB setting usually involve risks of hematomas or pseudoaneurysms formation at the access sites (femoral or radial), vascular dissections, or contrast-related complications (allergy or nephropathy), intestinal ischemia in LGIB, transient post-embolization syndrome, and symptomatic duodenal stenosis [19]. Risks of intestinal infarction post-IR-guided embolization therapy are usually minimal owing to the presence of well-established vascular anastomosis and collaterals within the gastrointestinal tract [20]. However, post-procedural gastric or duodenal infarction cases were reported in the literature secondary to the use of penetrating embolic agents like particulate PVA/liquid NBCA agents or in patients with previously damaged collateral vasculature networks from past abdominal surgeries and radiotherapy. Post-procedural complications were not seen in any of our patients. 

## Conclusions

Acute UGIB is a medical emergency and a common cause of hospital admissions worldwide. Refractory acute UGIB is associated with significant morbidity and mortality. TAE is a minimally invasive measure that should be considered early in the treatment of UGIB which is refractory to conventional endoscopic management.
